# Lymphangiomatous Polyp Presenting as Tonsillar Mass

**DOI:** 10.1155/2017/9506260

**Published:** 2017-10-15

**Authors:** Ashish Dhakal, Sameer Karmacharya, Sandhya Shrestha

**Affiliations:** ^1^Department of ENT-HNS, Dhulikhel Hospital, Kathmandu University Hospital, Dhulikhel, Kavre, Nepal; ^2^Department of Pathology, Dhulikhel Hospital, Kathmandu University Hospital, Dhulikhel, Kavre, Nepal

## Abstract

A 19-year-old female presented to ENT OPD of Dhulikhel Hospital, Kathmandu University Hospital, with history of foreign body sensation in throat for 2 weeks and mass in left tonsil for 1 week. There is no history of difficulty swallowing or recurrent throat infection. Physical examination revealed a pedunculated mass arising from upper pole of left tonsil. Bilateral tonsillectomy was done under general anaesthesia. Grossly, 2.5 × 1.5 × 1 cm polypoidal mass, soft in consistency, was found to be attached to left tonsil. Histopathology report was consistent with lymphangiomatous polyp of tonsil. Postoperative period was uneventful and she was normal during her follow-up at 1 month with bilateral healthy tonsillar fossa.

## 1. Introduction

Around 90% of all lymphatic malformations arise in head and neck [1, 2]. Most of them occur in skin and subcutaneous tissues. Other sites include larynx, parotid gland, mouth, and tongue [3].

Benign tumours or tumour-like lesions of palatine tonsil are more common than malignant ones. Squamous papilloma accounts for majority of benign lesions, whereas vascular tumours are rarely reported [4]. Tonsillar lymphangiomatous polyp is an uncommon hamartomatous lesion that generally arise from tonsillar surface, and it has rarely been reported in the medical literature [5].

## 2. Case Report

A 19-year-old female presented with history of foreign body sensation in throat for 2 weeks and mass in the left tonsillar region for 1 week. There is no history of sore throat or difficulty swallowing. There was no history of any chronic illness or similar illness in her family members.

Examination of oropharynx revealed a pedunculated mass arising from upper pole of left palatine tonsil. There was no cervical lymphadenopathy. Blood parameters were within normal level.

**Figure 1:**
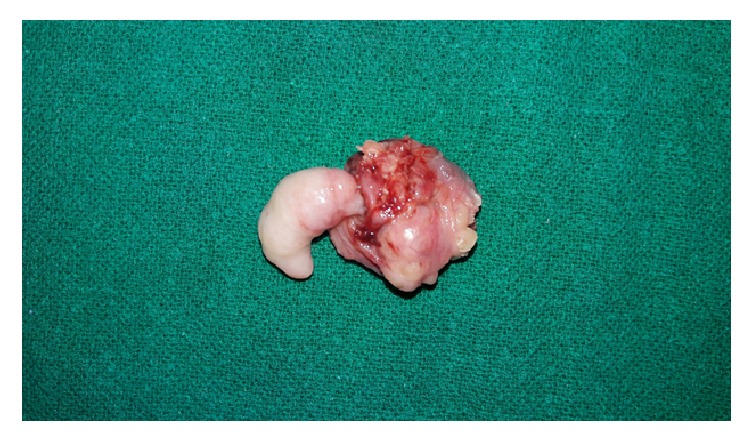
Gross photograph of left tonsillectomy specimen showing a polypoidal mass arising from upper pole.

Bilateral tonsillectomy was done under general anaesthesia. Grossly, a 2.5 × 1.5 × 1 cm, soft to firm, polypoidal mass was found attached to the upper pole of left tonsil with a slender stalk (Figure 1).

**Figure 2:**
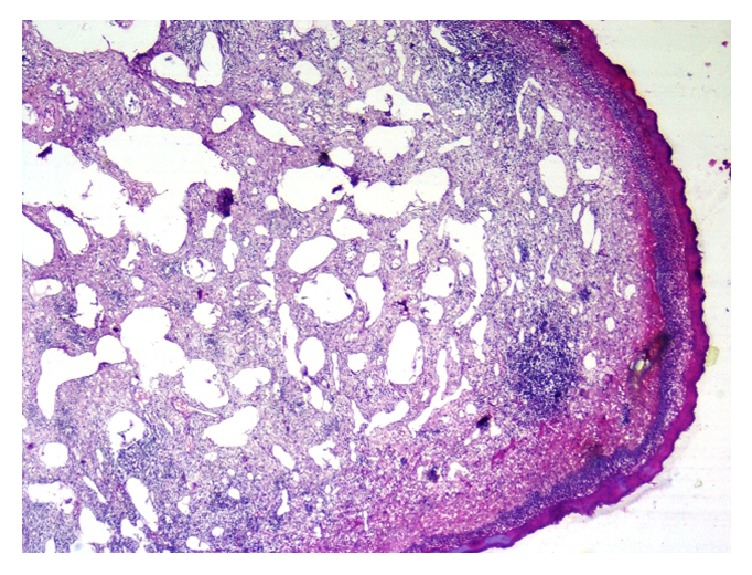
Histopathological examination showing tissue lined by stratified squamous epithelium lining with numerous dilated lymphatic channels present in underlying parenchyma (hematoxylin and eosin, 4x total magnification).

**Figure 3:**
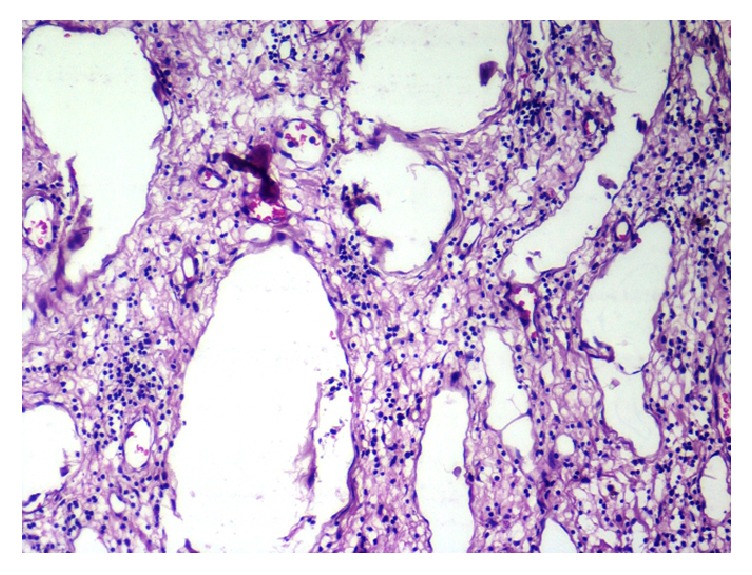
Histopathological examination showing dilated lymphatic channels lined by endothelium (hematoxylin and eosin, 10x total magnification high power).

The cut surface showed reddish areas. Histologically, the tissue was lined by hyperkeratotic, parakeratotic stratified squamous epithelium with underlying parenchyma showing numerous lymphatic channels lined by endothelium, some with luminal eosinophilic secretions. Intervening stroma was densely infiltrated by lymphocytes and plasma cells (Figures 2 and 3).

Postoperative period has been uneventful for 1 month of follow-up.

## 3. Discussion

Benign tumours are more common than malignant in case of palatine tonsils. Squamous papilloma accounts for the majority of benign lesions, whereas vascular tumours are rarely reported [6]. The tonsil is a less common site for the development of lymphatic malformations.

The true incidence of these types of tumour in the general population is currently unknown but many researchers suggest that it is most likely higher than expected [6, 7]. This is mainly thought to be due to lack of awareness of the tumour by the clinician and confusing histologic nomenclature use to describe benign lymphatic lesions in pathology literature [6]. In addition to this overlap in terminology, the nature of the tonsil, as a lymphoid organ rich in lymphocytes and efferent lymphatics, suggests that the lymphangiomatous polyps of the tonsil may not be as rare as it would seem and may be underrecognized and underreported [7, 8].

Pathogenesis of tonsillar lymphangiomatous polyp remains unclear. Chronic inflammation and associated obstruction of lymphatic channels were previously suggested as a possible mechanism, but this is unlikely given that chronic tonsillitis occurs much more commonly than lymphangiomatous polyp [6, 7].

Other mechanism like isolated hamartomatous proliferation is most likely responsible for the aetiology of these tumours. This is further supported by the haphazard proliferation of elements that are normally found in the tonsillar tissue [6, 8].

Lymphangiomatous polyps of the palatine tonsils have not been linked to other lymphatic lesions in the head and neck region nor do they seem to be part of generalized lymphatic malformation, which has been reported in the lower gastrointestinal tract [6, 8].

Mostly the management is tonsillectomy but some literatures have shown comparable result with excisional biopsy [9].

## 4. Conclusion

Lymphangiomatous polyps are uncommon benign lesion appearing in palatine tonsils. Often these are mistaken to be malignant growth causing unnecessary stress to the patient and their family. Tonsillectomy is the curative treatment in these conditions with no reported recurrence.
